# Domain-informed weight forecasting: leveraging behavioral and physiological sequences from wearables

**DOI:** 10.3389/fpubh.2026.1810381

**Published:** 2026-06-02

**Authors:** Luping Cheng, Lu Wang, Gaolei Wang, Bo Lu, Bei Wu, Yang Xiao, Qian Huang

**Affiliations:** 1 Department of Endocrinology, Shaanxi Provincial Traditional Chinese Medicine Hospital, Xi'an, China; 2 School of Management, Northwestern Polytechnical University, Xi'an, China

**Keywords:** body-weight prediction, digital health, feature engineering, LSTM, personalized medicine, wearable data

## Abstract

**Background:**

Short-term body-weight forecasting may support personalized weight monitoring, but many existing approaches rely on contemporaneous body weight or body mass index as model inputs, which limits practical use when frequent weigh-ins are unavailable.

**Methods:**

We developed a direct multi-step forecasting framework to predict 7-day body-weight trajectories from 14 days of behavioral, physiological, and lifestyle variables. The primary dataset was FitLife360, a synthetic longitudinal dataset on Kaggle, used for model development, benchmarking, and ablation. The LSTM forecaster was evaluated under a participant-level split, with subjects assigned to training, validation, or test sets before window construction. For real-world validation, we tested the framework on PMData from 16 participants over 5 months. Current body weight and BMI were excluded from model inputs and only used as targets. The LSTM was compared with Random Forest, and XGBoost.

**Results:**

On the synthetic FitLife360 dataset, the proposed LSTM achieved the best overall performance in the main comparison and showed consistent gains in the feature-ablation analysis. In the supplementary PMData experiment, the same framework remained operational on real-world wearable/lifelogging records, supporting the feasibility of the approach beyond the synthetic development setting. Detailed metrics for the supplementary experiment are reported in the main text.

**Conclusion:**

These findings should be interpreted primarily as evidence that domain-informed sequence modeling is feasible for short-horizon body-weight forecasting under both controlled synthetic and supplementary real-world data settings. However, such short-horizon predictions should not be interpreted as direct measures of meaningful adiposity change or short-term cardiometabolic risk, because day-to-day body weight also reflects transient physiological variability. The study therefore provides a methodological foundation for future validation in larger real-world and clinical cohorts.

## 1 Introduction

Excess body weight and adverse weight trajectories are major public health concerns worldwide. Obesity is a multifactorial condition shaped by behavioral, physiological, and environmental factors, and often co-occurs with other chronic conditions (1, 2). Beyond static body mass index (BMI), longitudinal weight dynamics provide additional descriptive information on individual variability and heterogeneous risk profiles (3).

At the population level, the burden of overweight and obesity continues to evolve across demographic groups and regions. Surveillance studies have documented shifting prevalence patterns, highlighting the need for scalable monitoring approaches beyond specialized clinical settings (4). Obesity-related metabolic abnormalities are also commonly observed in high-risk populations, with biomarkers such as serum uric acid associated with severe obesity phenotypes (5). These findings motivate the development of tools for tracking short-term weight-related changes.

With the rapid expansion of digital health, mobile applications, wearable devices, and AI-enabled platforms provide new opportunities to support weight monitoring beyond clinic-based models. Reviews suggest that such interventions can influence weight-related behaviors and outcomes, although their effectiveness varies across designs, populations, and levels of user engagement (6–10). From a measurement perspective, wearable devices enable continuous, low-burden collection of behavioral and physiological data streams, including diet, physical activity, and body weight (11). These systems are increasingly integrated into broader digital health ecosystems, incorporating multi-modal sensing and emerging data infrastructures (12–16).

Despite these advances, variability in adherence, data quality, and real-world usage remains a key challenge for translating digital health data into reliable insights. In addition, the digital environment shaping health-related behaviors has evolved, with social media influencing activity patterns and potentially affecting short-term weight dynamics (17). Digital health approaches have also been explored across various self-monitoring contexts, reflecting a broader shift toward technology-mediated behavioral tracking (18).

However, while monitoring has become easier, predicting near-term weight trajectories remains methodologically challenging. In the forecasting literature, approaches such as direct multi-horizon sequence models, hybrid correction schemes, and uncertainty-aware methods have been developed to improve multi-step reliability under noisy and non-stationary conditions (19–23). In contrast, many weight-related studies focus on long-term outcomes or categorical endpoints, rather than individualized short-term multi-step forecasting. Existing work includes early prediction of weight-change outcomes (24), digital health applications (25), and population-level risk modeling (26, 27), but these approaches do not directly address high-frequency, short-horizon forecasting of weight dynamics.

Furthermore, real-world behavioral and wearable data are often noisy, incomplete, and user-dependent, posing challenges for model robustness and validation (28). Short-term body mass fluctuations can also be non-trivial in certain contexts, requiring careful modeling and interpretation for short-horizon prediction (29).

Therefore, this study develops a time-series forecasting framework for short-term body-weight prediction using behavioral, physiological, and lifestyle-related variables commonly available in wearable and app-based datasets (12). The primary objective is methodological: to evaluate whether leakage-aware sequence modeling and domain-informed feature engineering can support short-horizon forecasting in a controlled longitudinal setting. To complement this, a supplementary experiment on a real-world dataset (PMData) is conducted to assess feasibility under real-world data conditions. The study should be interpreted as a proof-of-concept with supplementary validation rather than as direct evidence for clinical deployment (30).

## 2 Materials and methods

### 2.1 Study design and dataset

**Figure 1:**
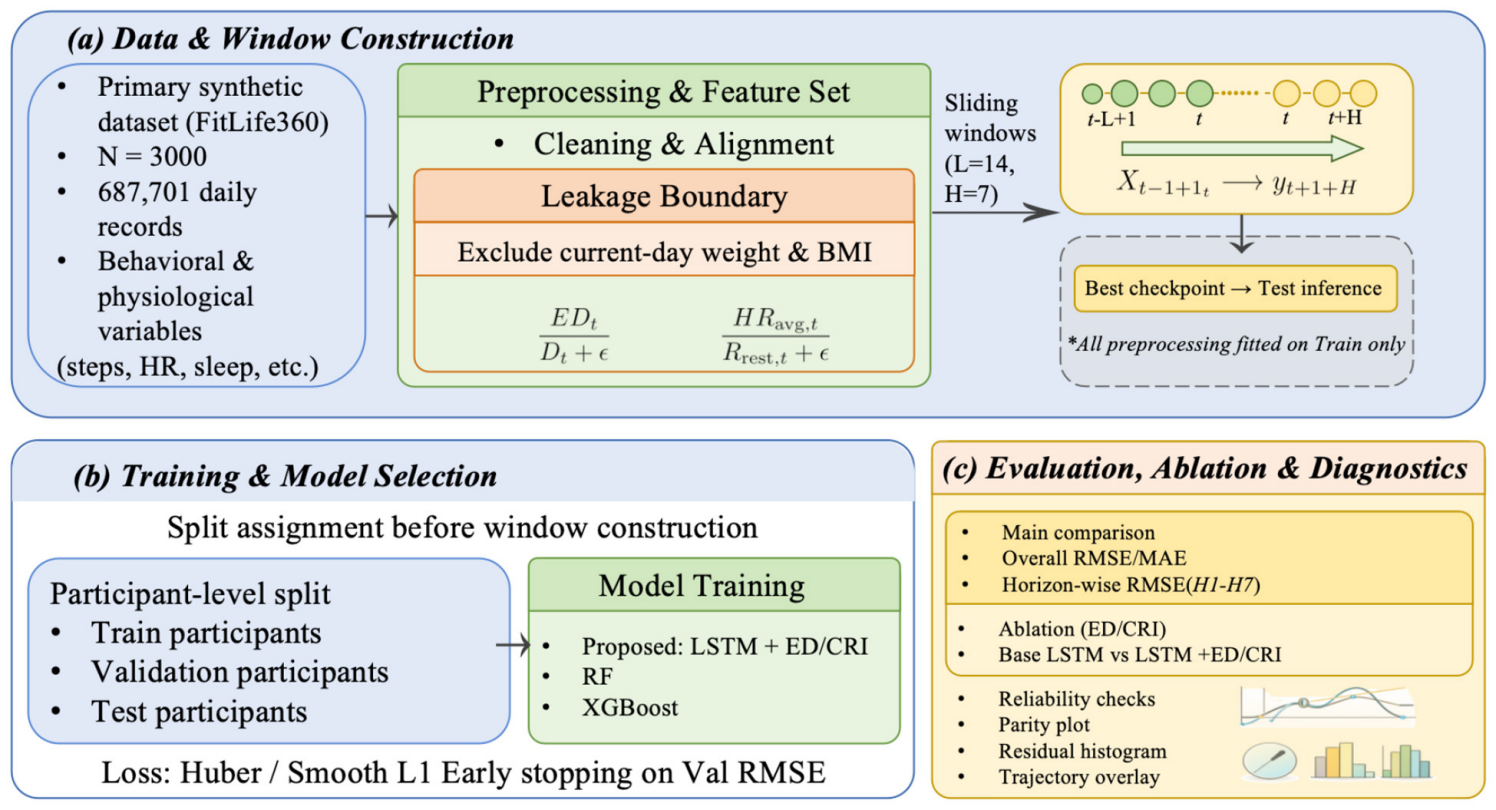
Leakage-aware workflow of the proposed short-horizon forecasting framework, including participant-level split assignment before sliding-window construction, preprocessing, model training, and multi-horizon evaluation. **(a)** Data and window construction. **(b)** Training and model selection. **(c)** Evaluation, ablation and diagnostics.

This study is a retrospective secondary analysis of publicly available datasets. The objective is to develop and evaluate a short-horizon forecasting framework for future body-weight trajectories using behavioral, physiological, and lifestyle-related variables, without using contemporaneous body weight or BMI as model inputs. Figure 1 summarizes the leakage-aware forecasting pipeline used in this study.

The primary dataset used for model development, benchmarking, and ablation was FitLife360, a publicly available dataset released on Kaggle.^1^ According to the repository description, FitLife360 is a *synthetic* longitudinal dataset simulating 1 year of health and fitness tracking records for 3,000 participants, including activity-, physiology-, and lifestyle-related variables. In this study, the dataset was used as a controlled benchmark for methodological development and evaluation (30).

To assess feasibility under real-world conditions, we additionally conducted a supplementary experiment using PMData.^2^ PMData is an open lifelogging and sports-logging dataset collected from 16 participants over approximately 5 months using Fitbit, Google Forms, and PMSYS. The PMData experiment was included as a supplementary real-world feasibility check rather than as a co-primary development benchmark.

Both datasets used in this study are publicly available and de-identified. This work involved only secondary analysis of open datasets containing individual-level records. No new human-subject data were collected, and there was no direct participant contact or intervention.

### 2.2 Outcome and input features

The prediction target (outcome) is *daily body weight* measured in kilograms (kg). We formulate the task as direct multi-step forecasting: given an input lookback sequence of length *L* = 14 days up to day *t*, the model predicts a vector of future body weights over a horizon of *H* = 7 days. For each supervised window, the forecasting formulation is given in Equation 1:


$$\begin{array}{l} X_{t-L+1:t}\in {\mathbb{R}}^{L\times d}\to {\hat{\text{y}}}_{t+1:t+H}\in {\mathbb{R}}^{H}, \end{array}$$ (1)

where X_*t*−*L*+1:*t*_ denotes the multivariate feature matrix over the past 14 days, *d* is the number of input features per day, and ${\hat{\text{y}}}_{t+1:t+H}$ denotes the predicted body-weight trajectory for the next 7 days. This study uses a *direct multi-output* setup, i.e., all seven future days are predicted simultaneously rather than recursively. Related work in multi-step time series prediction discusses hybrid strategies that combine recursive and multi-output designs, motivating careful horizon-wise evaluation even under direct prediction (31, 32).

To align with behavior-driven and proactive monitoring scenarios, current body weight and BMI were not used as input features. Body weight appears only as the supervised learning label (the future target sequence y_*t*+1:*t*+*H*_) and is excluded from the input feature set. BMI and any features that are direct transformations of current body weight (e.g., weight-derived rolling statistics or contemporaneous weight change) are likewise excluded. The anchor-day body weight *y*_*t*_ is retained only for diagnostics and baseline comparisons (e.g., persistence) and is *not* provided to the predictive model.

Daily input features were derived from behavior-, physiology-, and lifestyle-related variables represented in the two public datasets. For the primary FitLife360 analyses, these variables should be interpreted as synthetic wearable-/app-like proxies rather than audited device recordings. For the supplementary PMData experiment, the variables were derived from real-world lifelogging and wearable-related records. We used two types of inputs:

(i) Numeric features (standardized):
  - Demographic/anthropometric information. We used basic participant characteristics, including age and stature, to capture stable demographic and body-size differences across individuals.
  - Activity and exercise behavior. Daily physical activity was summarized using the total exercise duration, the estimated exercise-related energy expenditure, and step counts, which together reflect both structured workouts and overall mobility.
  - Cardiovascular status. We incorporated daily heart-rate and blood-pressure measurements (including average and resting heart rate, as well as systolic and diastolic pressure) to represent cardiorespiratory load and recovery.
  - Sleep and lifestyle indicators.s Sleep duration, perceived stress, hydration level, and self-reported fitness level were included as lifestyle-related proxies that may influence short-term weight fluctuations.
(ii) Categorical features (one-hot encoded with an explicit “unknown” category): gender, activity type, exercise intensity, self-reported health condition, and smoking status. Categorical variables were converted to indicator variables, and missing entries were retained as an explicit “unknown” category (rather than being imputed).

After encoding, the categorical block resulted in 27 dummy variables, yielding a base daily feature dimension of *d* = 13+27 = 40 (numeric + categorical). When handcrafted features ED and CRI were added (see below), the daily feature dimension increased to *d* = 15+27 = 42.

To summarize potentially relevant patterns in the wearable-derived activity and heart-rate signals, we engineered two domain-informed daily features and concatenated them to the input vector:

Let *E*_*t*_ denote the daily exercise-related energy expenditure (kcal) and *D*_*t*_ denote the total exercise duration (min) on day *t*, as recorded by the wearable/app logs in the dataset. We define Exercise Energy Density as shown in Equation 2:


$$\begin{array}{l} ED_{t}=\frac{E_{t}}{D_{t}+\epsilon }. \end{array}$$ (2)

ED serves as a heuristic, intensity-normalized summary of recorded exercise-related energy expenditure relative to exercise duration on day *t*. This type of intensity-normalization is consistent with broader training-load evaluation approaches that integrate energy expenditure, heart-rate, and movement-related signals (30, 33).

Let $HR_{t}^{\text{avg}}$ denote the daily average heart rate and $HR_{t}^{\text{rest}}$ denote the resting heart rate on day *t*, as provided by the wearable/app records in the dataset. We define the Cardiorespiratory Recovery Index as shown in Equation 3:


$$\begin{array}{l} CRI_{t}=\frac{HR_{t}^{\text{avg}}}{HR_{t}^{\text{rest}}+\epsilon }. \end{array}$$ (3)

CRI serves as a heuristic summary relating day-level average heart rate to resting heart rate. It is not intended as an established clinical index of recovery or cardiovascular function, but rather as a proxy-like ratio feature that may help summarize relative cardiovascular strain under the available wearable-derived measurements, with ϵ = 10^−8^. Resting heart rate is influenced by lifestyle factors and can reflect population-level fitness differences; meanwhile, wearable-based recovery monitoring has been investigated for identifying risk groups and tracking symptom-relevant changes (34, 35).

For both ED and CRI, division-by-zero was handled via the ϵ term; if a denominator was zero or missing in a given record, the resulting handcrafted feature for that day becomes missing and is treated consistently by the window filtering described below.

Numeric variables were *z*-score standardized using training-set statistics only, and categorical variables were one-hot encoded with a fixed dummy-column set learned from training to prevent leakage. We used strict complete-window filtering (no imputation): any window with missing values in the *L*-day inputs or *H*-day targets was excluded.

### 2.3 Model development and training

We formulated short-term body-weight forecasting as a direct multi-step sequence prediction problem. For each supervised sample, the model takes a multivariate behavioral and physiological feature sequence over a lookback window of length *L* = 14 days and predicts the future body-weight trajectory over a horizon of *H* = 7 days. Let ${\text{X}}_{t-L+1:t}\in {\mathbb{R}}^{L\times d}$ denote the input feature matrix for days *t*−*L*+1 to *t*, where *d* is the feature dimension after preprocessing and encoding. The target is the future absolute body-weight sequence, as shown in Equation 4:


$$\begin{array}{l} y_{t+1:t+H}\in {\mathbb{R}}^{H}. \end{array}$$ (4)

The forecasting task is therefore defined in Equation 5:


$$\begin{array}{l} X_{t-L+1:t}\to {\hat{\text{y}}}_{t+1:t+H}. \end{array}$$ (5)

This direct multi-output setup predicts all seven future steps simultaneously rather than recursively, thereby avoiding error accumulation across horizons.

We implemented an LSTM-based forecaster in PyTorch to capture temporal dependencies among behavioral and physiological variables. The encoder consists of a bidirectional LSTM followed by a multi-head self-attention layer. Given the input sequence X_*t*−*L*+1:*t*_, the bidirectional LSTM produces hidden representations as shown in Equation 6:


$$\begin{array}{l} H=\text{BiLSTM}\left({\text{X}}_{t-L+1:t}\right),\text{ H}\in {\mathbb{R}}^{L\times 2h}, \end{array}$$ (6)

where *h* is the hidden dimension in each direction.

A multi-head attention layer is then applied to the hidden states, followed by the residual connection defined in Equation 7:


$$\begin{array}{l} \tilde{\text{H}}=\text{H}+\text{MHA}\left(\text{H},\text{H},\text{H}\right). \end{array}$$ (7)

To summarize sequence-level temporal information, the final representation concatenates the mean-pooled representation and the last-step hidden representation, as shown in Equation 8:


$$\begin{array}{l} z=\left[\frac{1}{L}\overset{L}{\underset{j=1}{\sum }}{\tilde{h}}_{j};{\tilde{h}}_{L}\right]. \end{array}$$ (8)

The resulting representation is passed through a multilayer prediction head consisting of layer normalization, GELU activations, dropout, and fully connected layers to produce the *H*-step output trajectory given in Equation 9:


$$\begin{array}{l} {\hat{\text{y}}}_{t+1:t+H}=f_{\theta }\left(\text{z}\right). \end{array}$$ (9)

Model parameters were optimized using the Huber loss (Smooth L1 loss) with δ = 1.0, averaged across the forecast horizon, as defined in Equation 10:


$$\begin{array}{l} L=\frac{1}{H}\overset{H}{\underset{k=1}{\sum }}\ell_{\delta }\left(y_{t+k}-{ŷ}_{t+k}\right), \end{array}$$ (10)

where ℓ_δ_(·) denotes the Huber loss.

The model was trained using the AdamW optimizer with gradient norm clipping. Hyperparameters were selected according to validation RMSE from a predefined search grid, including hidden dimension {64, 128, 256}, number of LSTM layers {2, 3}, dropout {0.2, 0.3}, learning rate {10^−3^, 5 × 10^−4^}, and batch size {32, 64}. The weight decay coefficient was fixed at 10^−5^ and the gradient clipping threshold was fixed at 1.0.

Training was performed for up to 200 epochs. After each epoch, validation performance was evaluated using overall multi-horizon RMSE aggregated across all forecast steps. Early stopping was applied with a patience of 12 epochs based on validation RMSE. For each hyperparameter configuration, the checkpoint with the lowest validation RMSE was retained. The final model was selected according to the best validation RMSE and evaluated once on the held-out test set.

### 2.4 Model evaluation

We adopted a held-out evaluation protocol with separate training, validation, and test sets. To eliminate any possibility of cross-boundary overlap between lookback and forecast segments, participants were assigned to splits at the ID level before supervised sequence construction, and each participant belonged exclusively to one split.

Supervised samples were then generated independently within each participant using sliding windows with lookback *L* = 14 and forecast horizon *H* = 7. Only windows with fully consecutive daily observations across the entire *L*+*H* span were retained. Consequently, every valid window was fully contained within a single split, preventing both missing-data-induced discontinuity and cross-split leakage.

All preprocessing steps, including standardization statistics and one-hot dummy-column definitions, were fitted on the training split only and then applied unchanged to the validation and test splits. We used strict complete-window filtering without imputation: any window containing missing values in either the input or forecast segment was excluded. Anchor-day body weight *y*_*t*_ was used only for baselines and diagnostics and was never provided to the predictive models.

To contextualize multi-step forecasting performance, we compared the proposed LSTM forecaster with the following baselines:

- Random Forest (RF): the 14-day lookback window was flattened into a tabular input vector, and RF was trained as a direct multi-output regressor for the 7-step future weight trajectory.
- XGBoost: the same flattened lookback representation was used, and multi-step prediction was implemented using MultiOutputRegressor, i.e., one independent XGBoost regressor for each forecast horizon.

RF and XGBoost therefore served as static tabular multi-output baselines operating on flattened historical windows.

To quantify the incremental contribution of ED and CRI, we conducted a controlled ablation study by comparing:

- Base LSTM: behavioral and physiological inputs without ED/CRI;
- LSTM + ED/CRI: the same inputs augmented with Exercise Energy Density (ED) and Cardiorespiratory Recovery Index (CRI).

For strict comparability, the ablation analysis was performed on a matched subset of held-out test windows for which all inputs required by both variants were fully available and valid. Performance differences were therefore interpreted only within this same evaluation subset.

Performance was assessed on the held-out test windows using mean absolute error (MAE) and root mean squared error (RMSE) in kilograms. Let {*y*_*i, k*_} and {ŷ_*i, k*_} denote the ground-truth and predicted body weight for test window *i* at horizon step *k* (*k* = 1, …, *H*). We reported:

- Overall RMSE/MAE: aggregated across all forecast horizons and test windows,
$$\text{RMSE}=\sqrt{\frac{1}{N_{\text{test}}H}\overset{N_{\text{test}}}{\underset{i=1}{\sum }}\overset{H}{\underset{k=1}{\sum }}{\left(y_{i,k}-{ŷ}_{i,k}\right)}^{2}},$$
$$\text{MAE}=\frac{1}{N_{\text{test}}H}\overset{N_{\text{test}}}{\underset{i=1}{\sum }}\overset{H}{\underset{k=1}{\sum }}|y_{i,k}-{ŷ}_{i,k}|,$$
- Horizon-wise RMSE: computed separately for each forecast step to characterize error changes with forecast distance.

To quantify uncertainty under within-participant correlation, we additionally performed non-parametric bootstrap resampling at the participant level on the held-out test set. Specifically, test participants were resampled with replacement, and all test windows belonging to the sampled participants were retained for recomputation of overall RMSE and MAE. This procedure was repeated 1,000 times for each model, and 95% confidence intervals were obtained from the 2.5th and 97.5th percentiles of the bootstrap distribution.

**Figure 2:**
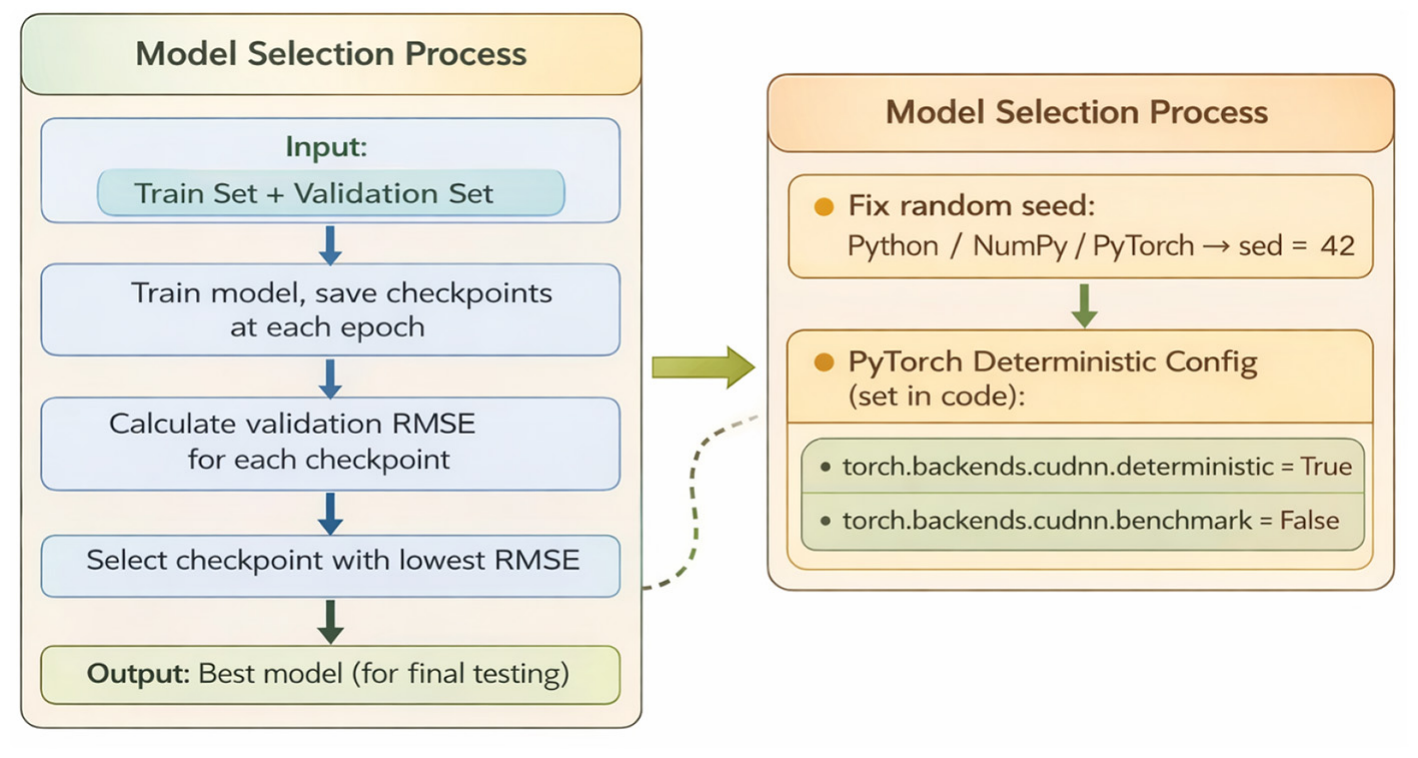
Model selection and reproducibility settings. The best checkpoint was selected by validation RMSE aggregated across horizons, and experiments were made reproducible through fixed random seeds and deterministic settings where applicable.

Model selection was based on validation RMSE aggregated across all forecast horizons. The checkpoint with the lowest validation RMSE was retained for final testing. To improve reproducibility, we fixed random seeds (Python/NumPy/PyTorch) and enabled deterministic computation settings where applicable. Figure 2 summarizes the checkpoint selection criterion and reproducibility configuration.

Beyond aggregate metrics, we generated diagnostic plots to assess prediction reliability and detect degenerate behaviors, including parity plots (true vs. predicted), residual histograms, and trajectory-level visual inspections. Such diagnostics are particularly important for wearable-derived behavioral data, which can be noisy and user-dependent (28, 30).

## 3 Results

### 3.1 Cohort and record characteristics

The primary analyses were based on FitLife360, a public synthetic longitudinal health and fitness dataset. The raw dataset contained 3,000 simulated participants and 687,701 daily records, with a median of 229 observed days per participant (IQR 223–235; range 198–261).

After preprocessing—including deduplication, removal of implausible adjacent-day weight jumps, segmentation by date continuity, and retention of continuous segments of at least 21 days—the analysis dataset comprised 221 participants and 5,416 daily records. In the retained sample, participants contributed a median of 24 observed days (IQR 22–26; range 21–54).

Using *L* = 14 and *H* = 7, we constructed 956 supervised sequence windows from the cleaned dataset for model development and evaluation under the main synthetic-data setting.

As a supplementary real-world validation resource, Because PMData is substantially smaller and less uniform than FitLife360, we used it as a supplementary feasibility check rather than as the main benchmark for model selection or large-scale ablation.

### 3.2 Exploratory feature association and implications

To contextualize feature engineering and model selection, we performed an exploratory feature association analysis. These analyses are *descriptive* and aim to characterize statistical associations in the observed data; therefore, they should *not* be interpreted as causal effects.

**Figure 3:**
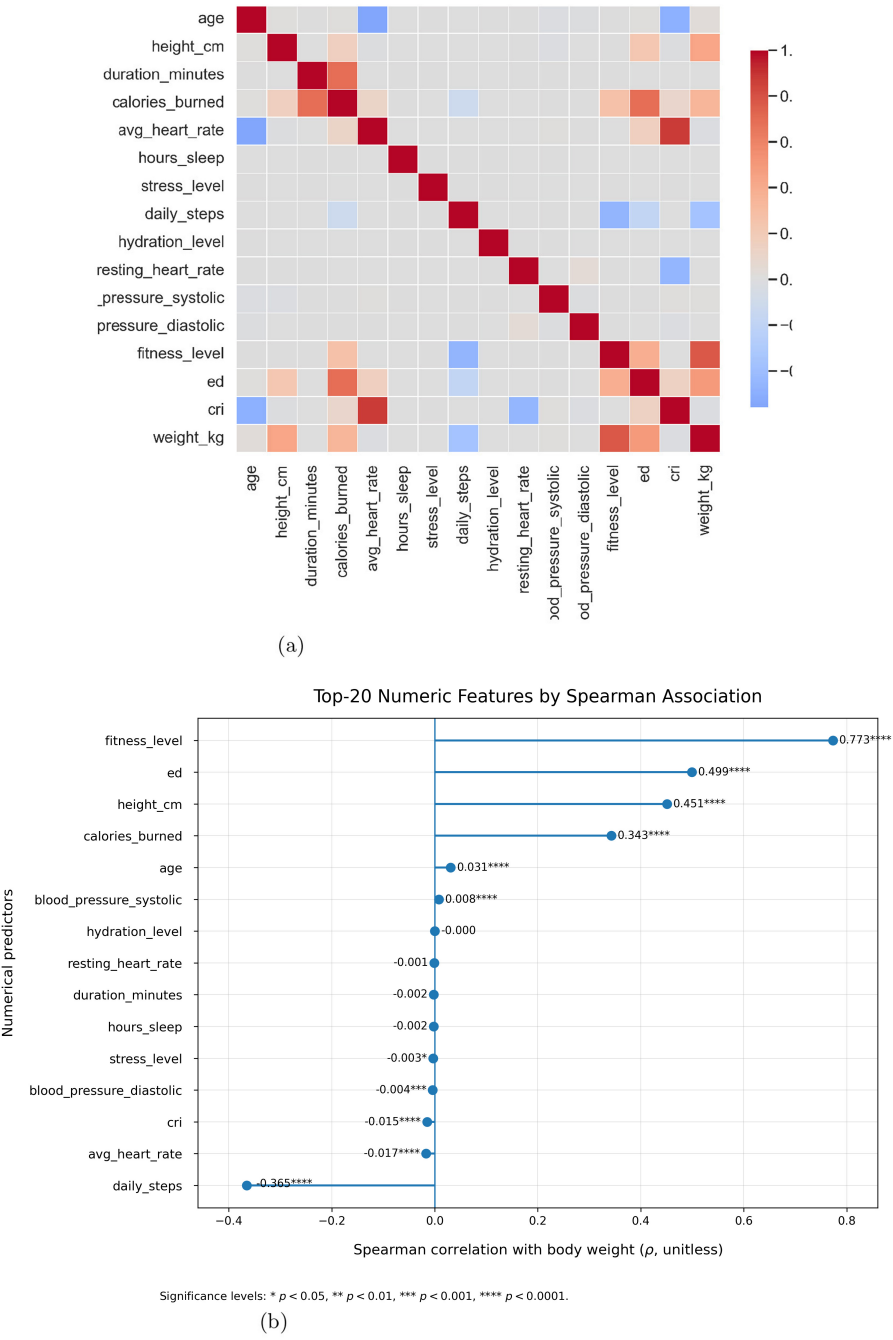
Exploratory associations between numerical predictors and body weight. **(a)** Spearman correlation heatmap. **(b)** Top-20 numerical predictors by |ρ|. Significance levels: *p < 0.05, **p < 0.01, ***p < 0.001, ****p < 0.0001.

For continuous variables, we quantified monotonic associations with body weight using Spearman's rank correlation coefficient, denoted as ρ. Figure 3a summarizes the pairwise correlations across numerical predictors and body weight, while Figure 3b highlights the variables with the strongest absolute correlations with body weight. Overall, most single-feature correlations were weak to moderate in magnitude, indicating that body weight is unlikely to be well-explained by any individual behavioral or physiological signal alone. Nevertheless, several features exhibited consistent directional associations, suggesting complementary predictive value when combined in a multivariate model.

**Figure 4:**
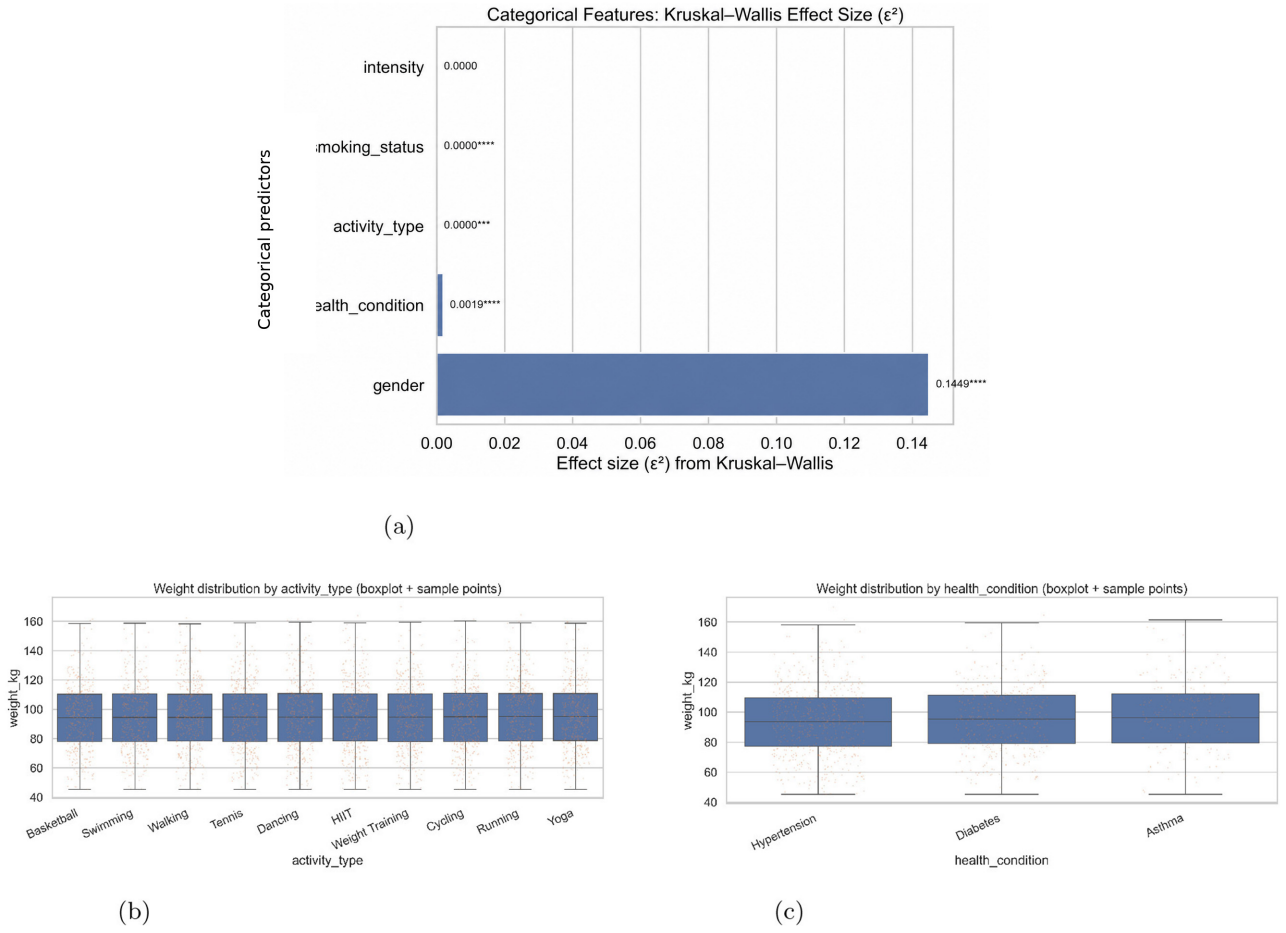
Exploratory associations for categorical predictors and representative group-wise distributions. **(a)** Effect sizes (ε^2^) from Kruskal–Wallis tests. **(b)** Body weight by activity type. **(c)** Body weight by health condition. Significance levels: *p < 0.05, **p < 0.01, ***p < 0.001, ****p < 0.0001.

For categorical predictors (e.g., activity type, intensity level, fitness level, smoking status, health-condition category), we evaluated differences in body weight distributions across groups using the Kruskal–Wallis test. To quantify practical significance beyond *p*-values, we reported the epsilon-squared effect size, ε^2^, as an interpretable non-parametric measure of between-group association, as shown in Equation 11:


$$\begin{array}{l} \varepsilon^{2}=\frac{H-k+1}{n-k}, \end{array}$$ (11)

where *H* is the Kruskal–Wallis test statistic, *k* is the number of groups, and *n* is the total sample size. Figure 4a provides an overview of ε^2^ across categorical predictors, and Figures 4b, c visualize representative group-wise distributions. In general, category-level differences were observable for several variables (e.g., fitness level), but effect sizes varied, reinforcing the need to integrate heterogeneous signals rather than relying on a single stratifier.

Overall, marginal associations were weak to moderate, suggesting that accuracy is unlikely to come from any single predictor and motivating multivariate, potentially non-linear modeling. Observed group-wise differences across categorical variables support explicit encoding. Because weight is temporally evolving, sequence modeling with historical context and domain-informed composite signals (e.g., ED and CRI) is better aligned with the data structure than static cross-sectional associations.

Because ED and CRI are ratio features derived from variables already present in the input set, their contribution should not be interpreted as introducing entirely independent physiological measurements. Rather, the exploratory correlation structure in Figure 3 suggests that these engineered features are closely related to their underlying components (ED to exercise duration and calories burned; CRI to average and resting heart rate). Accordingly, part of their benefit may arise from normalization, scale stabilization, and compression of correlated raw signals into lower-dimensional summaries, rather than from wholly new information.

### 3.3 Main predictive performance and model comparison

**Figure 5:**
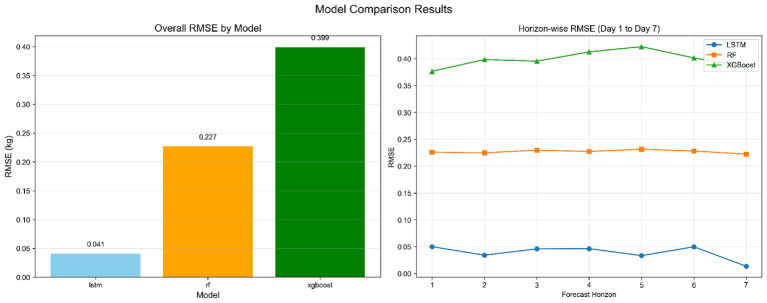
Model comparison results. **Left**: overall RMSE aggregated across all horizons. **Right**: horizon-wise RMSE for *H* = 7 days (day *t*+1 to day *t*+7).

We evaluated the proposed LSTM + domain-informed features (ED/CRI) model against two tabular-learning baselines: Random Forest (RF) and XGBoost. All models were assessed under the same participant-level train/validation/test split and leakage-aware preprocessing (11). Overall performance was reported using absolute-error metrics in kilograms (kg). Table 1 summarizes overall test performance aggregated across all forecast horizons (*H* = 7), while Figure 5 visualizes both overall RMSE and horizon-wise RMSE trends.

**Table 1:** Overall test performance comparison across models (aggregated over all horizons, units: kg).

Model	RMSE (95% CI)	MAE (95% CI)
LSTM (proposed, with ED/CRI)	0.041 (0.040, 0.042)	0.034 (0.033, 0.034)
Random Forest (RF)	0.227 (0.077, 0.369)	0.079 (0.045, 0.134)
XGBoost	0.399 (0.105, 0.719)	0.116 (0.070, 0.200)

To assess uncertainty under within-participant correlation, we further computed participant-bootstrap 95% confidence intervals for the overall test metrics. The proposed LSTM retained both the lowest point estimates and the narrowest uncertainty ranges [RMSE: 0.041 (0.040, 0.042); MAE: 0.034 (0.033, 0.034)], whereas RF and XGBoost showed substantially wider confidence intervals. This pattern suggests that the superior performance of the proposed model is unlikely to be explained solely by participant-level sampling variation under the present evaluation setting.

Across all forecast horizons, the proposed LSTM achieved the lowest prediction error, with an overall RMSE of 0.041 kg and MAE of 0.034 kg (Table 1). These results indicate that, under the present benchmark setting, the model was able to capture short-horizon temporal patterns in the longitudinal sequences. The tree-based baselines showed substantially larger errors (RF: RMSE = 0.227 kg, MAE = 0.079 kg; XGBoost: RMSE = 0.399 kg, MAE = 0.116 kg). This suggests that sequence-aware temporal modeling may provide advantages over static direct multi-output tabular baselines constructed from flattened historical windows in this forecasting setting (27, 28).

Figure 5 (right panel) and Table 2 report the horizon-wise root mean square errors (RMSE) from day t+1 to day t+7. The proposed LSTM model maintained consistently low error levels across all seven forecast horizons. In contrast, the horizon-wise RMSE curves of Random Forest (RF) and Extreme Gradient Boosting (XGBoost) remained relatively flat. This indicates that, under the current model specification, these static tabular baseline models possess limited horizon discrimination capability. From a digital-health research perspective, the ability to produce stable short-horizon forecasts from routinely collected signals may provide a useful methodological component for monitoring-oriented wearable frameworks (16).

**Table 2:** Horizon-wise RMSE (kg) for each model (day *t*+1 to *t*+7).

Model	H1	H2	H3	H4	H5	H6	H7
LSTM (ED/CRI)	**0.050**	**0.034**	**0.046**	**0.046**	**0.033**	**0.050**	**0.014**
RF	0.226	0.225	0.230	0.227	0.231	0.228	0.222
XGBoost	0.377	0.398	0.395	0.413	0.423	0.402	0.384

Bold values indicate the lowest RMSE, i.e., the best performance, within each forecast horizon.

The present results are most appropriately interpreted as methodological evidence that short-horizon body-weight trajectories can be forecast from behavioral, physiological, and lifestyle-related sequences under a leakage-aware evaluation protocol (36, 37). However, these trajectories should be understood primarily as near-term body-weight variability rather than direct markers of meaningful body-composition change. Under this framing, the practical value of the proposed framework lies in three aspects. First, it provides an individualized expected weight trajectory conditional on recent behavioral and physiological patterns, which may serve as a personalized reference for routine self-monitoring. Second, discrepancies between observed and expected short-term trajectories may help flag unusual or inconsistent patterns, such as transient dehydration, atypical fluid retention, abrupt behavioral disruption, or irregular measurement conditions. Third, within digital weight-management workflows, short-term forecasts may help contextualize normal day-to-day variability and reduce overinterpretation of routine fluctuations, thereby supporting more stable behavior-aware feedback and adherence monitoring (10).

### 3.4 Ablation and horizon-wise robustness

To isolate the contribution of the domain-informed engineered features, we compared: (i) a base LSTM trained using behavioral and physiological inputs only, and (ii) the proposed LSTM + ED/CRI variant. Both variants used the same window construction (*L* = 14, *H* = 7), participant-level train/validation/test split, and identical architecture, preprocessing, and optimization settings. Such feature ablation is useful for assessing whether engineered summary variables provide incremental predictive value beyond the original wearable-derived inputs (28).

Table 3 reports the test performance aggregated across all forecast horizons. Incorporating ED/CRI produced consistently lower errors than the base LSTM within the matched ablation subset. Specifically, overall RMSE decreased from 0.043 to 0.041 kg, while MAE decreased from 0.036 to 0.034 kg. These results suggest that the engineered ED/CRI features may provide complementary information beyond the original activity and cardiovascular variables under the present benchmark setting. However, because ED and CRI are constructed from variables already included in the input set, the observed improvement should be interpreted primarily as a feature-engineering effect rather than as evidence of entirely independent physiological information.

**Table 3:** Ablation results on the held-out test split (aggregated across all horizons, units: kg).

Model variant	RMSE (kg)	MAE (kg)
Base LSTM (wearable features only)	0.043	0.035
LSTM + ED/CRI (proposed)	**0.041**	**0.034**

Bold values indicate the lowest error, i.e., the best performance, within each metric column.

We further examined horizon-wise RMSE for each forecast step (*t*+1 to *t*+7) to assess prediction stability with increasing forecast distance. Table 4 shows that the ED/CRI variant achieved slightly lower errors across all forecast horizons within this ablation subset, while maintaining relatively stable multi-step performance across the 7-day horizon. At the same time, the magnitude of the improvement remained modest, and the stability observed here should be interpreted within the context of the controlled benchmark setting and strict continuity filtering protocol.

**Table 4:** Horizon-wise RMSE (kg) for ablation variants (day *t*+1 to *t*+7).

Model variant	H1	H2	H3	H4	H5	H6	H7
Base LSTM (no ED/CRI)	0.051	0.034	0.046	0.049	0.038	0.051	0.025
LSTM + ED/CRI	**0.049**	**0.034**	**0.046**	**0.046**	**0.033**	**0.050**	**0.025**

Bold values indicate the lowest RMSE, i.e., the best performance, within each forecast horizon.

Overall, the ablation results support the methodological usefulness of incorporating compact engineered summary features into sequence-based forecasting models under the evaluated benchmark setting. However, because the primary benchmark is synthetic and relatively regular after strict filtering, the observed gains should not be interpreted as direct evidence of real-world deployment effectiveness or clinical utility.

### 3.5 Qualitative and reliability checks

Accurate aggregate error metrics (RMSE/MAE) are necessary but not sufficient for evaluating short-term forecasting models in real-world digital health settings, where data can be noisy, user-dependent, and behavior-driven (7). Therefore, we conducted a set of qualitative and reliability checks to (i) verify that the proposed model does not exhibit degenerate behaviors (e.g., near-constant predictions), (ii) assess calibration and systematic bias across horizons, and (iii) ensure that performance gains are supported by well-behaved residual patterns. Given that consumer wearable outputs can vary in measurement validity, these checks complement aggregate metrics when working with real-world wearable-derived proxies (30).

**Figure 6:**
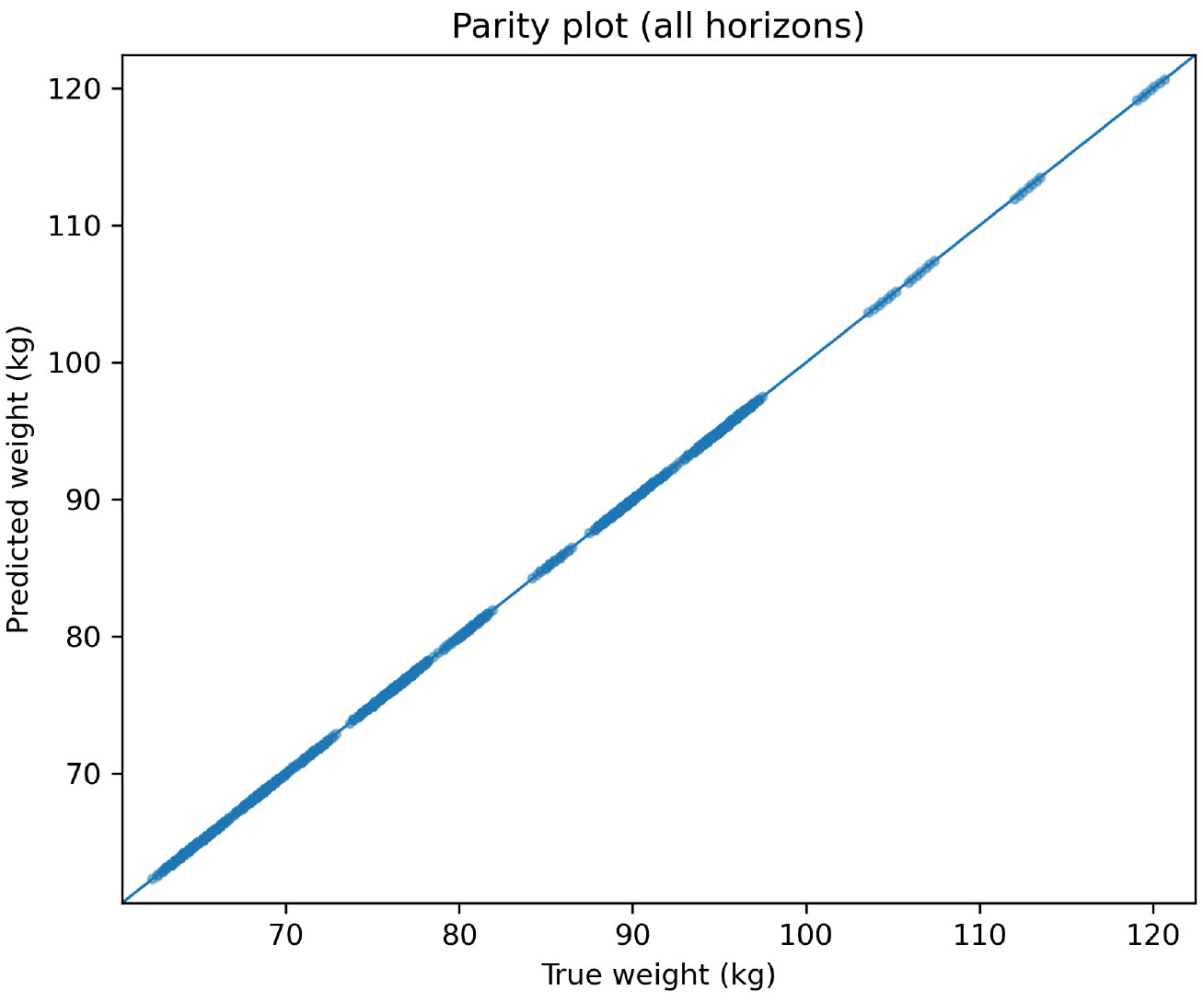
Parity plot aggregated across all horizons (*H* = 7). The concentration around the identity line suggests reasonable calibration and absence of degenerate constant-output behavior.

**Figure 7:**
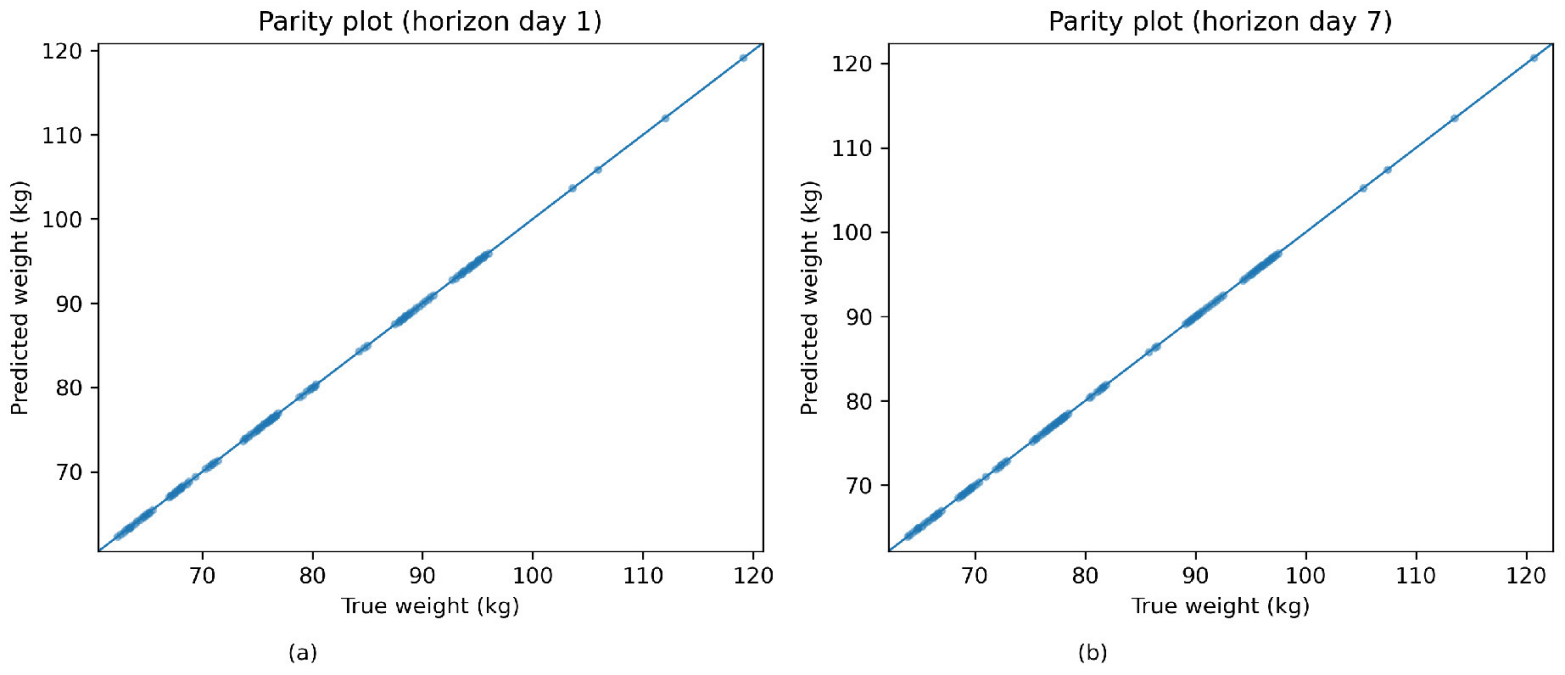
Horizon-specific parity plots for the proposed model: **(a)** horizon *H*1 (day *t*+1) and **(b)** horizon *H*7 (day *t*+7).

We first examined parity plots comparing predicted vs. observed body weight. Figure 6 shows the parity relationship aggregated across all horizons, and Figure 7 provides representative horizon-specific parity plots for *H*1 and *H*7. Overall, points concentrate around the identity line, indicating that the model produces predictions that are well-aligned with the observed scale. Importantly, the parity plots do not display the signature of “constant-output” collapse (i.e., a narrow vertical band of predictions independent of the ground truth), supporting that the model captures meaningful signal rather than regressing toward a trivial constant predictor. This is particularly important for short-horizon weight monitoring, where useful predictive signals should reflect day-to-day variation rather than only long-term averages (25).

**Figure 8:**
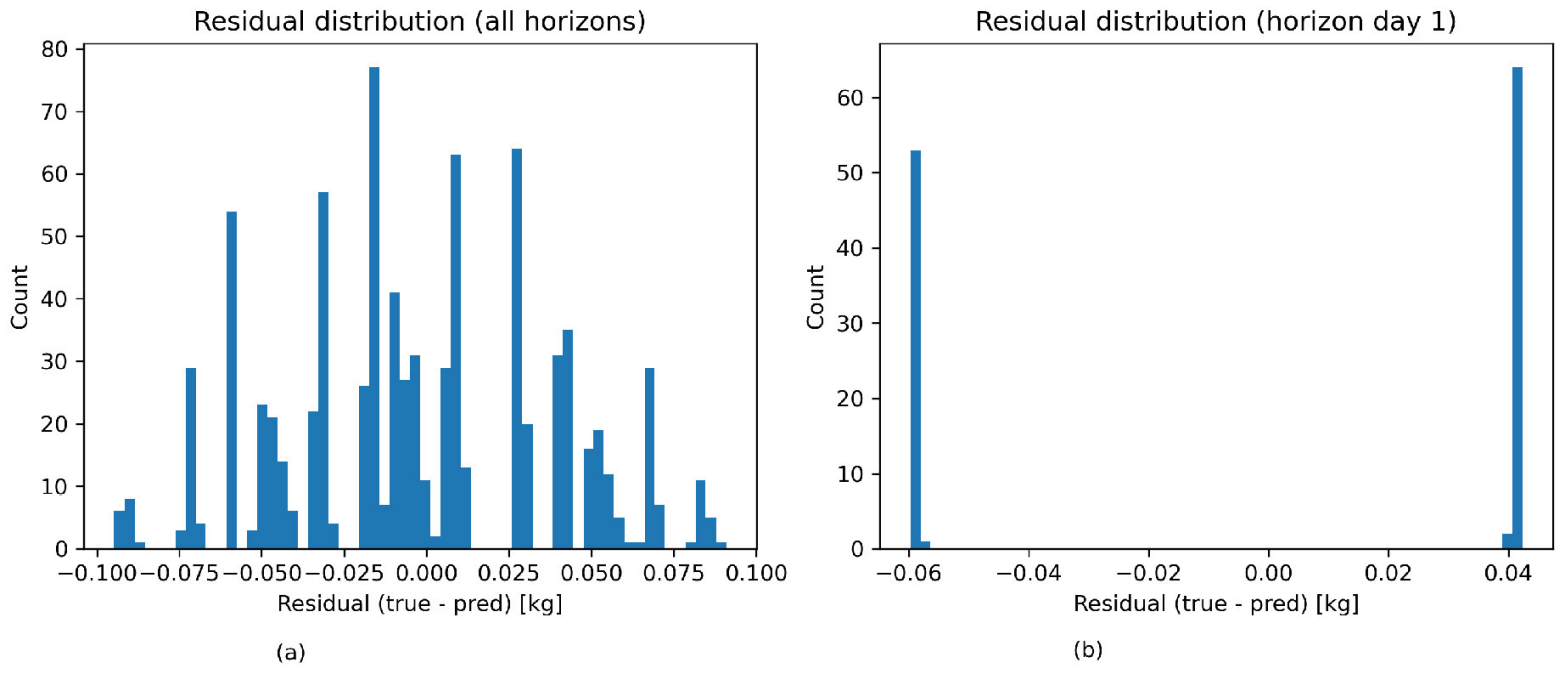
Residual distributions for the proposed model: **(a)** all horizons and **(b)** horizon *H*1 (day *t*+1).

We next analyzed residuals *e* = *y*−ŷ to detect systematic over- or under-estimation and to evaluate the stability of errors across horizons. Figure 8 presents the residual histogram aggregated across all horizons, while Figure 7 focuses on *H*1 as a representative short-term step. The residual distributions are centered close to zero and show no strong skewness, suggesting limited global bias. Moreover, the absence of heavy tails or multi-modal artifacts indicates that most predictions fall within a reasonable error band, consistent with reliable short-horizon monitoring requirements. Such residual-based checks are recommended when deploying predictive models on real-world lifestyle logs, where measurement noise and missingness can otherwise lead to misleadingly optimistic point metrics (26, 28, 38).

**Figure 9:**
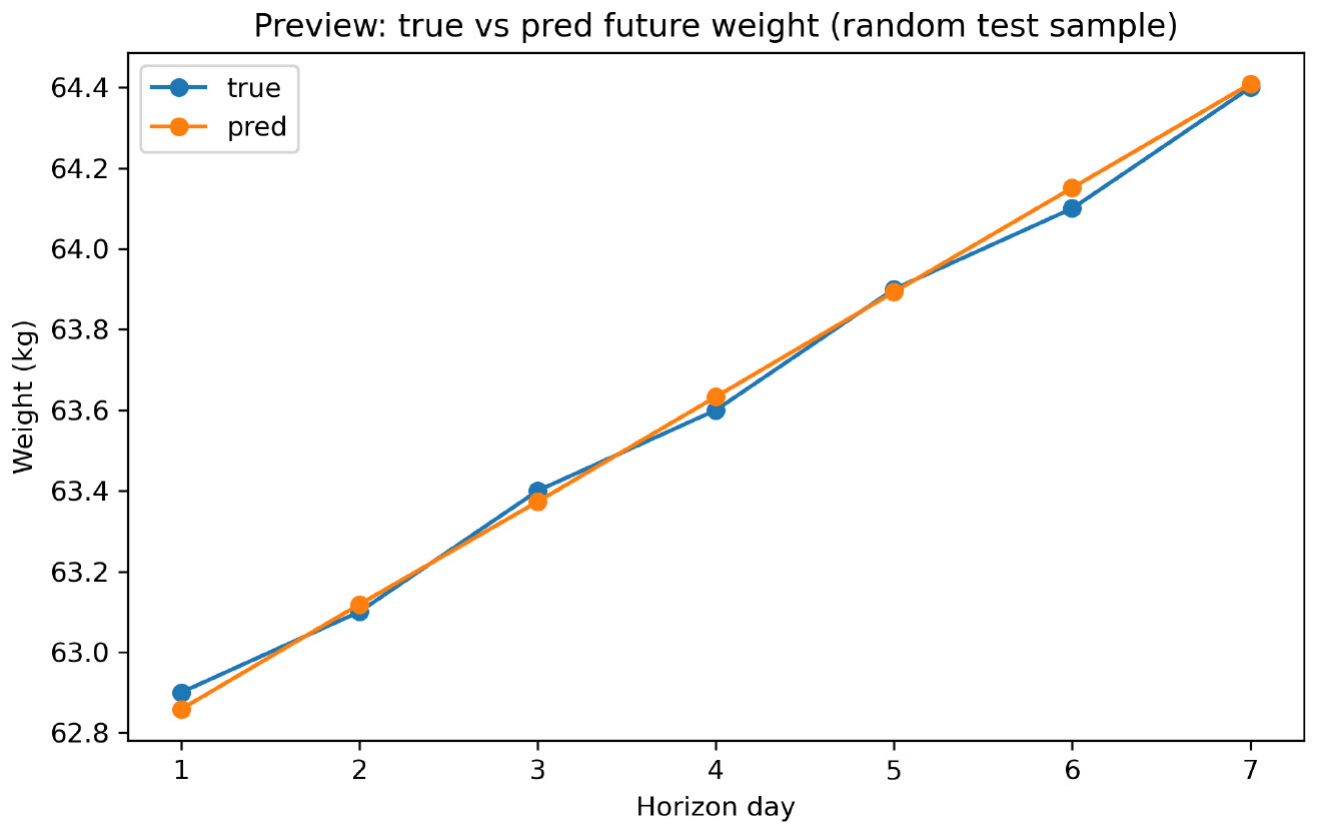
Representative qualitative example of predicted vs. observed short-term weight trajectory for a randomly selected test window.

In addition to pointwise diagnostics, we inspected predicted trajectories against observed trajectories for representative test windows to ensure that the model follows plausible temporal patterns. Figure 9 provides an illustrative example, showing that predicted curves track the general direction and magnitude of short-term changes without exhibiting unstable oscillations or unrealistic discontinuities. This trajectory-level inspection is relevant for digital health applications in which forecasts may serve as exploratory intermediate signals within broader decision-support systems, such as personalized monitoring platforms and wearable-integrated health management systems (14, 16).

Taken together, the qualitative and reliability checks support three conclusions: (i) the proposed model does not collapse to constant outputs and exhibits reasonable calibration in parity plots; (ii) residuals are centered near zero with no prominent skewness, indicating limited systematic bias; and (iii) predicted trajectories are visually plausible and stable, consistent with the goal of stable short-horizon forecasting within a digital health monitoring framework (7).

### 3.6 Supplementary real-world validation and ablation check on PMData

To complement the main analyses on the synthetic FitLife360 dataset, we conducted a supplementary experiment on PMData, a real-world lifelogging and sports-logging dataset. The purpose of this experiment was not to reproduce the scale of the synthetic benchmark, but to assess whether the proposed leakage-aware forecasting framework remains feasible under a smaller, noisier, and more heterogeneous real-world setting. This PMData experiment was included as a supplementary real-world feasibility and ablation check rather than as a co-primary benchmark.

After dataset-specific preprocessing and window construction on PMData, we compared two LSTM variants using the same overall forecasting logic as in the main study: (i) a base LSTM using wearable/lifelogging features only, and (ii) the proposed LSTM augmented with the domain-informed features ED and CRI. The quantitative results are summarized in Table 5.

**Table 5:** Supplementary real-world ablation results on PMData.

Model variant	Train RMSE	Train MAE	Test RMSE	Test MAE
LSTM + ED/CRI (proposed)	3.457	3.273	2.674	2.201
Base LSTM	1.928	1.635	2.807	2.267

The two variants exhibited different in-sample and out-of-sample behavior. The base LSTM achieved lower training error than the proposed model (train RMSE: 1.928 vs. 3.457; train MAE: 1.635 vs. 3.273), whereas the proposed LSTM+ED/CRI achieved lower error on the held-out test set (test RMSE: 2.674 vs. 2.807; test MAE: 2.201 vs. 2.267). This pattern suggests that, in the small and heterogeneous PMData setting, ED/CRI may improve out-of-sample generalization even though they do not improve in-sample fit.

These supplementary results should nevertheless be interpreted cautiously. PMData is much smaller than the synthetic development dataset and differs substantially in participant count, observation density, and measurement heterogeneity. Therefore, this experiment should be viewed as supportive real-world evidence for feasibility and potential generalization benefit, rather than as definitive proof of stable external superiority.

## 4 Discussion

### 4.1 Principal findings

This study developed a leakage-aware, direct multi-step forecasting framework for short-term body-weight prediction and evaluated it in two complementary settings: a primary synthetic longitudinal benchmark (FitLife360) for controlled model development, and a supplementary real-world lifelogging dataset (PMData) for practical feasibility assessment. In the synthetic development setting, the proposed LSTM + ED/CRI model achieved the best overall accuracy and stable horizon-wise performance. In the supplementary PMData experiment, the same framework remained feasible on real-world records, supporting the practical relevance of the approach while not eliminating the need for larger-scale external validation (28, 38). In the supplementary PMData experiment, the ED/CRI-augmented variant also achieved lower held-out test error than the base LSTM, providing additional small-sample real-world support for the potential generalization value of the engineered features.

### 4.2 Interpretive considerations for ED and CRI under noisy wearable measurements

Short-term body weight is influenced not only by slow changes in adipose mass but also by rapid fluctuations driven by *energy balance, glycogen and water, salt and hydration*, and *stress/sleep-related neuroendocrine responses*. Wearable-derived variables (steps, calories, heart rate, and sleep duration) capture parts of this latent physiology but are often noisy and device-dependent. In this context, ED and CRI should not be interpreted as entirely new physiological measurements; rather, they are ratio-based summary features derived from existing activity and heart-rate variables. Their value may therefore come partly from normalization and scale stabilization, while also encouraging the model to use more physiologically structured representations of exercise load and recovery status.

ED [ED_*t*_ = *E*_*t*_/(*D*_*t*_+ϵ)] normalizes estimated energy expenditure by activity duration and can therefore reflect differences between shorter higher-intensity activity and longer lower-intensity activity. Because short-term body weight may partly fluctuate with recent activity-related behavioral and physiological responses, ED may provide more informative short-horizon signals than raw calories or duration alone. More broadly, wearable-derived activity variables are often interpreted relative to exercise-load concepts while acknowledging the validity limitations of consumer devices (30, 33).

CRI [$\text{CR}{\text{I}}_{t}=HR_{t}^{\text{avg}}/\left(HR_{t}^{\text{rest}}+\epsilon \right)$] relates average daily heart rate to resting heart rate and can serve as a coarse proxy of relative cardiovascular strain. Although CRI is not a clinical recovery metric, elevated values may plausibly reflect lower recovery, stress, poorer sleep, or reduced fitness. These factors can in turn influence short-term behavioral and physiological states associated with body-weight fluctuation. Prior wearable studies have also explored resting heart rate and related recovery signals as responsive indicators of lifestyle and health status (39–41).

From a modeling perspective, ED and CRI compress correlated raw variables into more compact and scale-stabilized representations. Because both features are ratios, they partially reduce sensitivity to day-to-day variation in measurement scale and reporting patterns. Such normalization may improve signal consistency for short-horizon forecasting, where the model must rely on relatively subtle temporal variation. In this sense, ED and CRI can be interpreted primarily as domain-informed feature-engineering constructs rather than as independent physiological measurements.

### 4.3 Wearable signals as noisy proxy measurements

Recent wearable and accelerometry studies suggest that activity and heart-rate patterns can capture meaningful person-level behavioral variation associated with broader health trajectories (42–44). At the same time, consumer wearables remain heterogeneous in measurement quality, and variables such as energy expenditure are generally less reliable than simpler measures such as step count or heart rate (45, 46). Accordingly, the variables used in the present study should be interpreted as noisy proxy measurements rather than gold-standard physiological assessments. Within this context, ED and CRI are best understood as engineered summary features derived from wearable/app data rather than direct measurements of internal energy balance or clinical recovery status.

### 4.4 Relevance to digital health workflows

The findings of this study are more appropriately interpreted as methodological evidence relevant to digital health monitoring rather than direct evidence of clinical utility or population-level effectiveness. Because the primary benchmark was synthetic and the analyses focused on individual-level short-horizon prediction, the present results should be interpreted primarily as proof-of-concept forecasting evidence rather than deployment-ready clinical evidence. Within this narrower scope, models of this type could potentially serve as low-burden monitoring components in wearable-supported weight-management systems. In particular, an individualized predicted short-term trajectory may provide a reference against which observed body weight can be interpreted, helping distinguish routine day-to-day variability from more unusual short-term deviations. Such deviations may be useful for prompting re-checks of measurement conditions, reviewing recent behavior patterns, or providing more stable adherence-oriented feedback without overreacting to single-day fluctuations. These possible uses remain monitoring-oriented rather than diagnostic. We therefore do not claim that short-horizon prediction directly reflects clinically meaningful fat-mass change, intervention response, or immediate cardiometabolic benefit. Those questions require longer-horizon and metabolically anchored validation in future work.

### 4.5 Limitations and future work

Several limitations should be considered. First, the primary development benchmark (FitLife360) is synthetic rather than a genuine free-living wearable cohort. Although we added a supplementary PMData experiment, the present findings should still be interpreted mainly as methodological benchmark evidence rather than direct clinical or deployment-ready evidence. Second, short-term body-weight changes over 1–7 days may largely reflect transient physiological variation rather than meaningful changes in adiposity or cardiometabolic status. Therefore, the practical health value of short-horizon forecasting should be interpreted cautiously. Third, although participant-level splitting was adopted before window generation, sliding-window samples from the same participant remain correlated. Thus, the nominal number of windows should not be treated as the effective number of independent subject-level observations. Fourth, although participant-level bootstrap confidence intervals were added, uncertainty quantification remains limited to the present split and dataset. Future work should use larger cohorts, repeated re-splitting, and stronger external validation. Finally, ED and CRI are heuristic ratio-based proxy features derived from variables already included in the feature set. Their contribution may partly reflect normalization or collinearity-handling effects rather than entirely new physiological informat (47, 48).

## 5 Conclusion

This study presented a leakage-aware direct multi-step forecasting framework for short-term body-weight prediction using wearable-derived behavioral and physiological sequences, without using contemporaneous body weight or BMI as model inputs. The primary model-development analyses were conducted on FitLife360, a synthetic longitudinal dataset, where the proposed LSTM-based forecaster achieved stable held-out forecasting performance under the present benchmark setting and where ED/CRI provided modest improvements in the controlled ablation analysis.

To improve practical relevance, we additionally conducted a supplementary experiment on PMData, a real-world lifelogging and sports-logging dataset. Taken together, the results suggest that domain-informed sequence modeling may be a useful methodological direction for short-horizon weight forecasting in digital monitoring settings. Its value is better understood in terms of personalized reference-trajectory generation, deviation awareness, and behavior-aware feedback, rather than as a direct indicator of clinically meaningful fat-mass change or immediate cardiometabolic outcomes.

Future work should prioritize larger real-world wearable cohorts, stronger external validation, participant-level uncertainty quantification, and subgroup-specific robustness analysis.

## Data Availability

The original contributions presented in the study are included in the article/supplementary material, further inquiries can be directed to the corresponding authors.

## Footnotes

1
https://www.kaggle.com/datasets/jijagallery/fitlife-health-and-fitness-tracking-dataset

2
https://datasets.simula.no/pmdata/
